# Resilient agri-food systems for nutrition amidst COVID-19: evidence and lessons from food-based approaches to overcome micronutrient deficiency and rebuild livelihoods after crises

**DOI:** 10.1007/s12571-020-01067-2

**Published:** 2020-07-25

**Authors:** Simon Heck, Hugo Campos, Ian Barker, Julius J. Okello, Arun Baral, Erick Boy, Lynn Brown, Ekin Birol

**Affiliations:** 1 International Potato Center, Nairobi, Kenya; 2 International Potato Center, Lima, Peru; 3 International Potato Center, Basel, Switzerland; 4 International Potato Center, Kampala, Uganda; 5 HarvestPlus, Washington, DC USA

**Keywords:** COVID-19, nutrition, biofortification, humanitarian crisis, food systems, potato, sweetpotato, innovation, jobs to be done theory

## Abstract

COVID-19 has had an instant effect on food systems in developing countries. Restrictions to the movement of people and goods have impaired access to markets, services and food. Unlike other concurrent crises, rather than threatening the material hardware of food systems, COVID-19 has so far affected the ‘software’ of food systems, highlighting again that connectivity is at the heart of these systems. Drops in demand, the loss of markets and employment and growing concerns about international cooperation are indications of possible deeper disruptions to come. Amidst this uncertainty, strategies to safeguard food and nutrition security of the world’s poor need to prioritize diversification of production and markets. Nutritious, biofortified crops such as potato, sweetpotato, but also wheat, maize and beans among others, can play a more significant role to provide key micronutrients (vitamin A, iron, zinc) at large scale. Strong local market chains, robust smallholder production systems and increasing commercial utilization make these crops powerful vehicles for securing nutrition when markets and mobility look uncertain. We posit that the evolving impacts of COVID-19 provide an opportunity to focus agricultural innovations, including the development and delivery of biofortified crops, on new and more specifically defined ‘jobs to be done’ throughout the food system. This will help bridge some of the current disruptions in supply and demand and will help prepare food systems for future crises.

## Introduction

The impact of COVID-19 on food systems was felt almost immediately. Restrictions to movement of people and goods meant that initially physical and soon economic access to markets, and to food, became disrupted irrespective of the pandemic’s impact on food production. Wheat and rice prices have already jumped by 8% and 25% compared to March 2019, respectively (Torero 2020). In this initial phase, COVID-19 has so far been a different kind of crisis from those sparked by natural disasters like the droughts, floods, cyclones and pests hitting the food systems of Africa and Asia over the past decades. COVID-19 has entered food systems through the disruption of markets, institutions and the workings of social capital that woven together are so important for the resilience of food systems. This is being particularly felt by the world’s poor who rely more heavily on the informal sector and thus on daily economic and social transactions to maintain their livelihoods and get access to food.

Rather than threatening the ‘material hardware’ of food production, as other concurrent climatic and ecological crises do, COVID-19 has so far affected the ‘software’ of food systems. During the first three months, it has been a crisis of disrupted connections between supply and demand, even within seemingly well-established supply chains. Loss of markets and employment have left many millions of farmers and food sector workers wondering when and in what form their income opportunities will come back. In the longer term, through weakened economies, massive unemployment, increased draw on public sector resources, and greater uncertainty of international cooperation, COVID-19 might also lead to a wider crisis of food and nutrition security for some of the world’s most vulnerable populations, in rural and urban communities. What are the implications of this kind of crisis for strategies to build more resilient food systems that can serve the priorities of the world’s poor? Specifically, how can food systems keep generating nutrition and livelihood benefits for these vulnerable populations under COVID-19 conditions?

In this paper we reflect on how agricultural innovations can help meet these challenges, drawing specifically on experiences from utilizing potato, sweetpotato and the broader biofortification approach to improve nutrition and livelihoods during previous crises, and identifying key knowledge gaps emerging from COVID-19.

## Potato and sweetpotato – insights from crisis responses

Given their comparative advantage as high yielding and fast-growing crops with strong local market demand, and rather insulated from international price spikes, potato and sweetpotato have frequently been used as ‘crisis response crops’ following natural disasters in Africa and to some extent elsewhere. These crops also contributed to intensification and diversification of local food systems otherwise dominated by cereals, as in Asia, and thus helped strengthen their ability to withstand and recover from shocks (Prain and Naziri 2020). Evidence shows that farmers were able to quickly establish potato and sweetpotato fields once they received planting material and technical backstopping through emergency campaigns, and to maintain production for several years without further subsequent support. Their short growing cycles makes food, and feedstuff, available to affected households and communities faster and earlier in the season. Drought and disease tolerant varieties make harvests more reliable also under more marginal agro-ecological conditions. Surplus sales provide cash income that can be spent in meeting immediate food and non-food household needs (Okello et al. 2019). Moreover, these crops are naturally nutritious, and through breeding, the content of micronutrients has been increased, such as vitamin A in sweetpotato, and iron in potato. The former is already reaching millions of households in Africa, and the latter is at an advanced stage of development (Hannele Lindqvist-Kreuze, International Potato Center, *pers. comm*.). Research shows that households that plant improved varieties of these crops obtain higher yields and revenues, are less food insecure, and have more diverse diets and a better health status (Okello et al. 2017; Jones and de Brauw 2015; De Brauw et al. 2018; De Brauw et al. 2019). 

In some cases, such emergency interventions provided an opportunity to accelerate the introduction of improved and more nutritious varieties at large scale, such as in Mozambique where nutritious biofortified sweetpotato varieties have been disseminated by Government and relief agencies during recurrent hunger relief programs over the past 10 years. As a result, about one third of Mozambique’s sweetpotato today are vitamin A biofortified, making significant contributions to vitamin A consumption among rural populations with otherwise poor access to markets and health services (Jenkins et al. 2018). Similarly, the political unrest of 2008/09 in Kenya spurred the entry of the private sector into the supply of quality seed of improved potato varieties which carried important pro-poor traits. Likewise, the epidemics of maize lethal necrosis from 2012 onwards and fall army worm from 2018 in East Africa resulted in increasing production of alternative crops such as beans and potatoes as intercrops or part of a wider rotation (One Acre Fund 2019). These short cycle crops, which have always contributed to filling the hunger-gap on-farm before the maize harvest, went on to contribute to improved food security and nutrition at the national scale.

Turning crisis into opportunity for increasing access to nutritious crops, albeit through one-off interventions, can kickstart lasting change toward greater food and nutrition security where other efforts may have taken much longer. At field level, coordinated efforts to accelerate delivery of productive inputs and information are more likely during crises, and at policy level locally important crops receive greater attention from policy makers who otherwise are focused on a few internationally traded commodities. On the other hand, and relating these lessons to COVID-19, analyses also show that expanding access to larger populations and providing a pipeline of improved varieties over time also requires investment in the ‘software’ of local food systems (Worstell 2020). Creating connections to markets and institutional resources has been essential for sustaining the food security and nutrition benefits from these emergency responses. Supporting sustainable seed enterprises through technical training and facilitating their access to technologies and markets is an important element of this strategy. Experiences from Mozambique and Ethiopia illustrate this. Channeling recurrent emergency responses in Mozambique through a network of public and private sector seed multipliers built up local capacities for producing quality sweetpotato planting material over time that could be activated in 2019 to respond almost immediately to the food emergency following cyclone Idai. Within only six months, 14,000 new households in the worst hit areas were able to grow improved, climate resilient biofortified sweetpotato varieties (International Potato Center 2019a). In Ethiopia, during the 2016 drought, 10,000 households quickly received potato and sweetpotato seed through local suppliers accompanied by additional information on improved seed management to strengthen their capacity for dealing with future shortfalls (International Potato Center 2019b).

Seed enterprises can thus become multi-functional, trading seed and information for a range of crops, and contributing directly to diversification and hence resilience of local food systems. These rural enterprises and their networks of suppliers and customers can also be effective partners in future crisis responses, providing sources of seed and food for humanitarian efforts closer to locations of need and thus linking food or seed deficit areas to suppliers in the same country. COVID-19, at least in the short term, poses additional constraints at an operational level through restrictions to movement and assembly that require changes to how goods and services are being delivered. Already, the innovation capabilities and economies of scale of the ICT sector have started to transform the world’s food systems (World Bank 2019). Bringing this capacity to bear on the needs of smallholder farmers and poor consumers at the bottom of the pyramid should be relatively easy with the right partnerships.

## Safeguarding nutrition through biofortification of staple crops

Biofortification, the enrichment of commonly consumed staple crops with nutrients such as vitamin A, iron, and zinc, has proven to be an efficacious, acceptable and cost-effective solution for reducing deficiencies in these micronutrients, and in improving various health outcomes (Bouis & Saltzman 2017; Bouis et al. 2019; Birol & Bouis 2019). These benefits are critical both during the COVID-19 humanitarian crisis and its aftermath. Research has shown that the amounts of these micronutrients found in healthy, diversified diets have a beneficial effect on the human immune system including its ability to counter some types of viruses (Wessels et al. 2017; Read et al. 2019). An estimated 2.5 billion people are at risk of micronutrient deficiencies (Saltzman et al. 2017) as their diets do not meet their biological requirements for these micronutrients. This ‘hidden hunger’ is expected to increase as a result of disruptions to the global food systems due to COVID-19 (see United Nations System Standing Committee on Nutrition) if lessons from the global food price crisis of over a decade ago also apply today (Christian 2010). While important differences in the nature of these crises and their likely effects on food systems have been pointed out, some key parallels exist that pose threats to nutrition and micronutrient supplies in particular.

An immediate negative impact of COVID-19 has been the decline in household incomes, especially for the majority of the poor in LMICs who work in the informal sector. This is further compounded by the sharp decline in remittances to LMICs already projected by the World Bank for 2020, which further impairs the food purchasing power of vulnerable populations. Lower household incomes often translate into shifts in food consumption patterns, increasing reliance on staples to meet caloric needs (Bouis et al. 2011) and lowering consumption of more expensive, nutrient dense animal source foods, fruits and vegetables. This trend may be further exacerbated during COVID-19 in countries where disruptions to transportation and restricted mobility of farm labor jeopardizes supply chains of perishable horticultural crops and animal source foods thus increasing their prices and putting them further out of reach of poor families.

Restrictions may have similar effects on the delivery of other important micronutrient supply strategies. Industrial fortification of commonly consumed foods (such as flour, oil and sugar) is widely effective in reaching consumers also in urban markets, but may also become disrupted should supplies of fortificants, factory operations, inspection and support services, transportation and marketing of fortified products be affected. Initiatives in the health sector, such as Vitamin A supplementation programs, may also be disrupted at least in the short run through shifts of resources toward COVID-19 testing and treatment, and through constraints from social distancing rules for program delivery. In sum, falling incomes, market disruptions, increasing prices of nutrient-dense foods, and shifting policy priorities caused by COVID-19 are likely to increase the importance of biofortified staples as a reliable, fast and affordable source of micronutrients.

Biofortification could help address some of the long-term concerns about building ‘micronutrient resiliency’ among food and nutrition insecure populations in expectation of future pandemics or other crises. Where biofortified crops are well integrated in local food production systems and reliably and affordably deliver key micronutrients alongside basic calories, this resiliency will be strengthened. Great progress is being made in achieving the potential of biofortification, specifically through accelerated development and deployment of new varieties. Almost 350 biofortified varieties of 11 key staples are released for production in 40 countries globally (see this map of which biofortified crops are available where). Over the past 10 years, the work by the CGIAR’s HarvestPlus and CIP and their partners has enabled an estimated 15 million households to grow biofortified crops, including orange-fleshed sweetpotato, across Africa, Asia and Latin America.

At the same time, COVID-19 raises new questions and opportunities for scaling up biofortification to reach much larger populations, both farmers and consumers, in many more countries. Since biofortified staple crops are produced and consumed locally (if not on farm) and since several of them (such as vitamin A maize, iron beans and pearl millet, and zinc rice, maize and wheat) tend to have long shelf lives, their integration in food systems would provide micronutrient resiliency for farming households and local consumers whose purchasing power and access to markets is impaired as a result of pandemics. This micronutrient resiliency is also likely to improve consumers’ resilience to potential future pandemics given the aforementioned proven impact of micronutrient status on human immune function. As a result of COVID-19, HarvestPlus and CIP have turned to digital technologies and strategies avoiding physical contacts for reaching farmers and consumers with information about the benefits, availability and sources of biofortified seeds and foods, as well as accelerated partnerships with public and humanitarian sectors, for delivery of both biofortified seeds and food to populations most vulnerable to food and nutrition insecurity. As they pivot the delivery models for biofortified seeds and food due to COVID-19, CIP and HarvestPlus are implementing a rigorous learning agenda around these innovations to better understand their cost-effectiveness, equity, impact and scalability aspects so as to be even better prepared for the next crisis.

## The job to be done: Directing agricultural innovation to meet the needs of the poor

Looking ahead, while we cannot foresee the depth, length and nature of the food and nutrition security fallout from COVID-19, we should take the current uncertainties as a further reminder of how much is at stake for the world’s poor and how much we still have to learn about addressing their needs through the rich set of technologies and knowledge we have developed. It starts with identifying the ‘job to be done’, the problem to be solved from the perspective of those whose problem it is, in this case the poor that are vulnerable to recurrent crises. The ‘job to be done’ theory, developed at Harvard Business School, provides a customer-driven framework for innovation which puts the needs of the customer at its center (Christensen et al. 2016). It has been implemented in many innovation efforts of both private and public sectors. Campos (2020) makes a strong case for applying this perspective to addressing agricultural and food systems challenges and lays out why this is a non-trivial first step when trying to solve familiar but wicked problems, more so under conditions of uncertainty. A preliminary analysis of the COVID-19 food systems implications so far suggests several ‘jobs to be done’. As noted widely, restrictions from the pandemic response in several countries have led to short term disconnects between consumers without physical access to food and producers without marketing outlets. This has affected in particular the marketing of perishable nutritious foods. Expecting that such disruptions may re-occur with future waves and new pandemics, there may be need to diversify market supply chains to ensure that such foods can be sourced from shorter distances or can be stored safely and economically in a processed form that still meets the food preferences of consumers. More needs to be understood about supply chain and technology options for ‘stabilizing’ the supply of key nutrients from nutrient-dense foods, including biofortified crops and horticultural produce, to low-income consumers through diverse market chains of processed and fresh products. A ‘job to be done’ perspective will caution us not to prescribe solutions off the shelf, but co-create options with consumers, retailers, service providers and farmers. Addressing the needs and satisfaction gaps of end-users becomes the goal and starting point of agricultural innovation - also when combating undernourishment and micronutrient insecurity.

A second, and more fundamental market disruption from COVID-19 has been the decline of purchasing power among many millions of already poor consumers following their loss of jobs and incomes. This has often been compounded by their relocation from familiar social and economic environments around their places of employment for an undefined length of time. At this time, it is unclear how deep and long this disruption will be and how it will affect food access for different categories of these consumers in different market segments and market contexts. This uncertainty raises the issue of scale and agency when prioritizing among potential response strategies. From the consumer’s perspective, the ‘job to be done’ will most likely center around securing reliable and affordable access to familiar food items though they might be willing to compromise on food selection under restricted circumstances. For the farmers, traders and retailers, the problem to be solved at this time will have more to do with timing of harvests, selling through existing supply chain agreements, and managing stockpiles – all in a manner that minimizes losses and potentially creates opportunities for incremental gain from shifting consumer demand. From a public sector viewpoint, the ‘job to be done’ will however include concerns about social stability and nutrition and health risks from this decline in purchasing power that includes large number of young wage earners, both women and men, with children under five and two years of age.

## Conclusion

Niels Bohr’s quote ‘Prediction is very difficult, especially about the future’ appears more relevant than ever. Nevertheless, building from previous experiences and incorporating novel perspectives such as ‘the job to be done’, it is possible to conceive how to mitigate the consequences of this humanitarian crisis: In the very short term (weeks to months), it is essential to keep food flowing and to enable migrant workers to move and access health checks. This will help stabilize food prices and markets to continue operating. To achieve this, policy makers need to carefully consider the far-reaching consequences of export control measures and restrictions in regional cross-border trade. In the short term (next 12 months), farmers require reliable access to seed and other productive inputs so they can produce the food and feed they need to support their families and sell their surplus. Biofortified crops can make a particular contribution in this regard. In the mid to long term (next 2 years), food systems need to invest in diversification and connectivity to manage increased interdependence as core strategies for building resilience to the ‘unknown unknowns’ lying ahead.

Crises such as the Covid-19 pandemic and others whether natural or man-made seem to drive both technological and system changes that foster innovation and hopefully continually build resilience against future shocks. As an example, we are already seeing Kenyan private rose growers turning their capacity and knowledge to producing potato apical rooted cuttings for sale to local seed producers when faced with the loss of their export trade. Diverting even a small proportion of the production capacity of the very capable East African export flower and vegetable sector to local food production may in the future build resilience for both the exporters, local seed producers and for food security in general.

If COVID-19 has so far been a crisis of disrupted connections, it is also a reminder that connectivity between people and resources lies at the heart of functioning food systems which in turn are essential to our ability to cope with global crises; or in John Boyd Orr’s words, “We cannot build peace on empty stomachs”.
